# Ability of the ISAS3Fun Method to Detect Sperm Acrosome Integrity and Its Potential to Discriminate between High and Low Field Fertility Bulls

**DOI:** 10.3390/biology10111135

**Published:** 2021-11-04

**Authors:** Jesús L. Yániz, Inmaculada Palacín, Miguel A. Silvestre, Carlos Olegario Hidalgo, Carolina Tamargo, Pilar Santolaria

**Affiliations:** 1 BIOFITER Research Group, Institute of Environmental Sciences (IUCA), University of Zaragoza, 22071 Huesca, Spain; ipalacin@unizar.es (I.P.); psantola@unizar.es (P.S.); 2 Departamento de Biología Celular, Biología Funcional y Antropología Física, Universidad de Valencia, 46100 Burjassot, Spain; miguel.silvestre@uv.es; 3 Animal Genetics and Reproduction Area, Regional Agrifood Research and Development Service (SERIDA), 33394 Gijón, Spain; cohidalgo@serida.org (C.O.H.); ctamargo@serida.org (C.T.)

**Keywords:** *Bos taurus*, spermiogram, male fertility, capacitation, acrosome reaction

## Abstract

**Simple Summary:**

The objective of the present study was to investigate whether differences in bull fertility are associated with variations of sperm quality. Differences between high- and low-fertility bulls were found mainly in parameters related to sperm acrosome integrity when using a new fluorescence method that allows clear and precise detection of the sperm plasma membrane and acrosome: the ISAS3Fun method. It was concluded that the simultaneous assessment of sperm viability and acrosome integrity with the ISAS3Fun method is precise and seems to have a greater potential to discriminate between high- and low-fertility bulls than the more conventional in vitro sperm quality test. These results may help to predict the breeding soundness of bulls used in artificial insemination, which is important for the dairy industry.

**Abstract:**

The objective of the present study was to investigate whether fertility differences in bulls are reflected in variations of sperm quality when analysing only one ejaculate per male. Two experiments were performed. In the first experiment, frozen semen samples from 20 adult bulls were tested; 10 bulls had high field fertility and 10 bulls had low field fertility. Analyses of sperm motility, membrane integrity, and membrane–acrosome integrity with the ISAS3Fun method were performed. Sperm morphometry of the fluorescence sperm subpopulations obtained with the ISAS3Fun method was also analysed. Significant differences between high- and low-fertility groups were only found with the ISAS3Fun technique, specifically in sperm acrosome integrity, the proportion of spermatozoa with an intact acrosome and damaged membrane, and in sperm head width of spermatozoa with intact structures. Discriminant analyses allowed us to correctly classify 90% of sperm samples in their fertility group using sperm quality parameters. Given that only the results obtained with the ISAS3Fun technique were related to bull fertility, we performed a second experiment aimed to validate the efficacy of this technique to detect the acrosomal integrity of bull spermatozoa, comparing them with the conventional FITC-PNA/propidium iodide (PNA/PI) combination under capacitating conditions. The results indicated that the ISAS3Fun combination provided an accurate assessment of both viability and acrosomal integrity for ejaculated spermatozoa, while the PNA/PI combination underestimated the extension of acrosomal damage due to false negatives. It was concluded that the simultaneous assessment of sperm plasma membranes and acrosome integrity with the ISAS3Fun method is precise and seems to have a greater potential to discriminate between high- and low-fertility bulls than more conventional in vitro sperm quality tests.

## 1. Introduction

The prediction of breeding soundness of bulls and of the fertilising ability of semen used in artificial insemination (AI) is important for the dairy industry [1]. However, achieving satisfactory predictions based on the in vitro evaluation of sperm quality remains a challenge and still heavily relies on the simple assessment of sperm motility and morphology [2]. Beyond these important parameters, a motile sperm may not be able to fertilise an oocyte if it does not have an intact acrosome or may give rise to a viable embryo if its DNA is damaged. After spermatogenesis, bull sperm appears to be quite resistant to DNA fragmentation [3], but the acrosome is a delicate membranous structure that can be severely damaged during freezing and thawing procedures, which determines the success or failure of AI [4].

The development of acrosome-specific fluorescent probes, among which lectins are the most widely used, has facilitated the determination of acrosomal damage, even using flow cytometry. *Pisum sativum* (edible pea) agglutinin (PSA) and *Arachis hypogaea* (peanut) agglutinin (PNA) are the most commonly used lectins for this purpose [5]. They bind to glycosidic residues in different parts of the acrosomal membrane and, being conjugated with a fluorochrome such as fluorescein isothiocyanate (FITC), sperm with damaged acrosomes may be easily visualised. Nevertheless, extensive acrosomal damage can produce false negatives due to missing binding sites. [5]. The use of acidotropic probes, with an affinity for organelles such as the acrosome, has been shown to be ineffective due to non-specific (LysoSensor^TM^ Green DND-189) or heterogeneous (LysoTracker^®^ DND-99 and dapoxyl 2-aminoethyl sulphonamide) labelling [5]. Although the pH of the acrosome is acidic, it gradually increases during the course of sperm capacitation [6].

In previous studies [7,8], we described a fluorochrome combination (ISAS3Fun) that allowed us to discriminate sperm viability, acrosomal integrity, and sperm functionality in bulls. This study was designed to investigate whether differences in the field fertility of bulls were reflected in variations of sperm quality parameters, including those obtained with the ISAS3Fun test, when analysing only one ejaculate per male. Given that only the results obtained with the ISAS3Fun test were related to bull fertility, we conducted a study in which the efficacy of this technique was compared with the conventional FITC-PNA/PI (PNA/PI) combination to detect the acrosomal integrity of bull spermatozoa before and after incubation under capacitating conditions and the induction of an acrosome reaction.

## 2. Materials and Methods

Unless otherwise indicated, all chemicals were obtained from Sigma (Merck Life Science S.L.U., Madrid, Spain).

### 2.1. Semen Collection and Processing

This study analysed cryopreserved semen samples collected from commercial Holstein–Friesian bulls. Ejaculates with a total sperm motility or with morphologically normal spermatozoa lower than 70% were discarded. The ejaculates were extended in BullXcell (IMV Technologies, Humeco, Huesca, Spain) to a final concentration of 1.3 × 10^7^ sperm per 0.25 mL semen straw (IMV Technologies). All straws were cryopreserved following standard procedures [9]. Before use, straws were thawed for 1 min at 37 °C in a water bath and processed.

### 2.2. Experimental Design

To achieve the proposed objectives, two experiments were performed.

**Experiment 1:** Relationship between in vitro sperm quality test and field fertility of bulls.

Frozen semen samples from 20 bulls, 10 of high field fertility and 10 with low field fertility, were selected for this experiment with an age range between 2 and 6 years. Field fertility was determined by a retrospective study using data from the first and second AI postpartum results of at least 100 Holstein–Friesian cows in their second lactation over a period of 5 y (2009 to 2014) in different herds. Then, 353 bulls were evaluated and, between them, the 10 bulls with the highest (>53%) and 10 bulls with the lowest (<39%) fertility were included in the study. Cow fertility (pregnancy rate) was determined through pregnancy diagnosis using ultrasonography or rectal palpation of the genital tract at 35–45 days post-AI. Two frozen samples from the same ejaculate of each bull were collected between those used for the field fertility analysis. Semen samples were thawed, pooled, and processed for sperm quality assessment as detailed below.

#### 2.2.1. Sperm Motility (CASA-Mot)

Within a few minutes after thawing, a diluted semen sample was placed in a pre-warmed Makler chamber (Sefi-Medical Instruments Ltd., Haifa, Israel), and a computer-assisted sperm analyser (CASA-Mot; ISAS^®^, version 1.32; PROISER, Paterna, Spain) was used to assess sperm motility [10]. Spermatozoa were classified as progressive if VCL > 20 μm/s and STR > 80%.

#### 2.2.2. Sperm Plasma Membrane Integrity

Sperm plasma membrane integrity (viability) was determined using an acridine orange and propidium iodide combination [11]. The number of total spermatozoa and the percentages of membrane-intact spermatozoa were determined using the OpenCASA software [12]. At least 300 sperm cells were analysed per sample.

#### 2.2.3. Sperm Plasma/Acrosome Membrane Integrity

**Figure 1:**
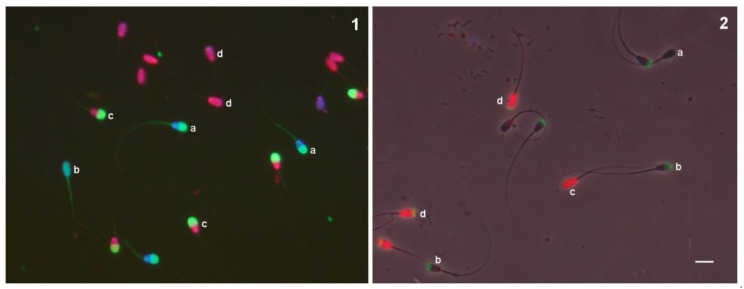
Fluorescence image of bull spermatozoa stained with ISAS3Fun kit (**1**) and fluorescence-phase contrast image of bull spermatozoa stained with PI/PNA. (**2**): a, Intact plasma membrane and acrosome; b, intact plasma membrane and damaged acrosome; c, damaged plasma membrane and intact acrosome; d, damaged plasma membrane and acrosome. Scale bar = 10 µm.

Combined sperm plasma/acrosome membrane integrity was determined using the ISAS3Fun technique (PROISER), as described in a previous work [8]. The labelling mix included propidium iodide (PI), Hoechst 33342, and carboxyfluorescein diacetate (CFDA). Two hundred spermatozoa were examined on each slide, and the staining patterns were classified into four categories (Figure 1), aided with v2 of the OpenCASA software [12]: (a) those with intact acrosomes and plasma membranes (IAIM); (b) those with intact acrosomes and damaged plasma membranes (IADM); (c) those with damaged acrosomes and intact plasma membranes (DAIM); and (d) those with damaged acrosomes and plasma membranes (DADM) [7].

#### 2.2.4. Sperm Morphometry (CASA-Morph)

Sperm head morphometry of the fluorescent sperm subpopulations was determined as described in [7]. Briefly, spermatozoa were stained with the fluorochromes of the ISAS3Fun kit, and the morphometry of the sperm head, nucleus, and acrosome of the different fluorescent subpopulations was determined using a specific plug-in module created on ImageJ.

**Experiment 2:** Validation of the use of the ISAS3Fun method for the assessment of sperm acrosome integrity in bulls.

Frozen semen samples from 18 bulls were thawed in a water bath, centrifuged (600× *g*, 10 min), and resuspended in S-TALP (sperm Tyrode’s albumin, lactate, and pyruvate) medium [13] with 10 IU/mL sodium heparin salt to induce sperm capacitation. The spermatozoa were allowed to capacitate for 4 h at 39 °C under 5% CO_2_. Ionomycin (7 μM final concentration) was added to the sperm suspension to induce the acrosome reaction, and the incubation period was extended for 1 h in the same conditions.

Aliquots were evaluated at 0 h, 4 h (after induced capacitation), and 5 h (an hour after the induced acrosome reaction) for sperm motility with the CASA system and for sperm plasma/acrosome membrane integrity as detailed below.

For each incubation time, a sample aliquot was mixed in a vial with PNA (10 μg/mL) for 10 min and PI (15 μM) for 5 min at 37 °C. Then, 3 μL of the labelled sample was placed on a prewarmed slide, covered, and pressed with the Trumorph^®^ system [14]. Stained spermatozoa were examined under a fluorescence/phase contrast microscope (DM4500B, Leica, Wetzlar, Germany) equipped with a warmed stage and a 20× plan apochromatic objective, and a standard green/red filter set (G/R, excitation: 490–520, 575–630 nm) was used to obtain digital images of the fluorescence-labelled sperm. The staining patterns of the spermatozoa were first examined using fluorescence microscopy, and then the localisation was confirmed using phase contrast optics. Two hundred spermatozoa were examined on each slide, and the staining patterns were classified into four categories (Figure 1): (a) those unstained (intact acrosome and plasma membrane, IAIM); (b) those with stained nuclei (intact acrosome and damaged plasma membrane, IADM); (c) those with a stained acrosome (damaged acrosome and intact plasma membrane, DAIM); and (d) those with a stained acrosome and nuclei (damaged acrosome and plasma membrane, DADM).

Another sample aliquot was labelled with the ISAS3Fun kit (PROISER), as detailed above.

### 2.3. Statistical Analysis

Statistical analyses were performed using the SPSS package, version 23.0 (SPSS Inc., Chicago, IL, USA). Statistical significance was set at *p* < 0.05. Distribution normality and the homogeneity of variance of the median score for each dataset were checked using the Kolmogorov–Smirnov and Levene tests, respectively. As data were normally distributed, parametric analysis was performed throughout. In the first experiment, differences in the sperm quality results between high- and low-fertility bulls were evaluated by Wilk’s lambda ANOVA. A discriminant analysis was also performed with the linear stepwise procedure to identify the most useful parameters for the classification of high- and low-fertility bulls. Wilk’s lambda ANOVA was used to compare the fraction of the total dispersion of data not accounted for.

In the second experiment, the effects of time (0 h, 4 h, and 5 h) using the ISAS3Fun method vs. the PNA/PI combination on the sperm membrane and acrosome integrity (IM and IA, respectively), as well as on the distribution of sperm subpopulations (IAIM, DAIM, IADM, and DADM), were analysed by General Linear Models (GLM) repeated measures analysis of variance.

## 3. Results

### 3.1. Experiment 1

Significant differences between high- and low-fertility bulls were observed in acrosome integrity, the proportion of IADM sperm and the head width of the IAIM sperm subpopulation (Table 1). Highly fertile bulls had a higher acrosome integrity, percentage of IADM spermatozoa, and head width of the IAIM subpopulation (*p* < 0.05) than those of low-fertility bulls. No statistical differences were found for the other sperm quality parameters analysed.

A discriminant analysis was performed to determine if it was possible to distinguish to which group of bull fertility each individual semen sample belonged. The results indicated that the percentage of the IADM sperm subpopulation, the head width, and the acrosome perimeter of the IAIM sperm subpopulation were the variables selected as the best discriminators of high and low field fertility. The matrix of classification obtained gave the Fisher’s discriminant linear functions for each class (Table 2, *p* < 0.001), with a globally correct assignment of 90% of the samples (Table 3).

### 3.2. Experiment 2

**Figure 2:**
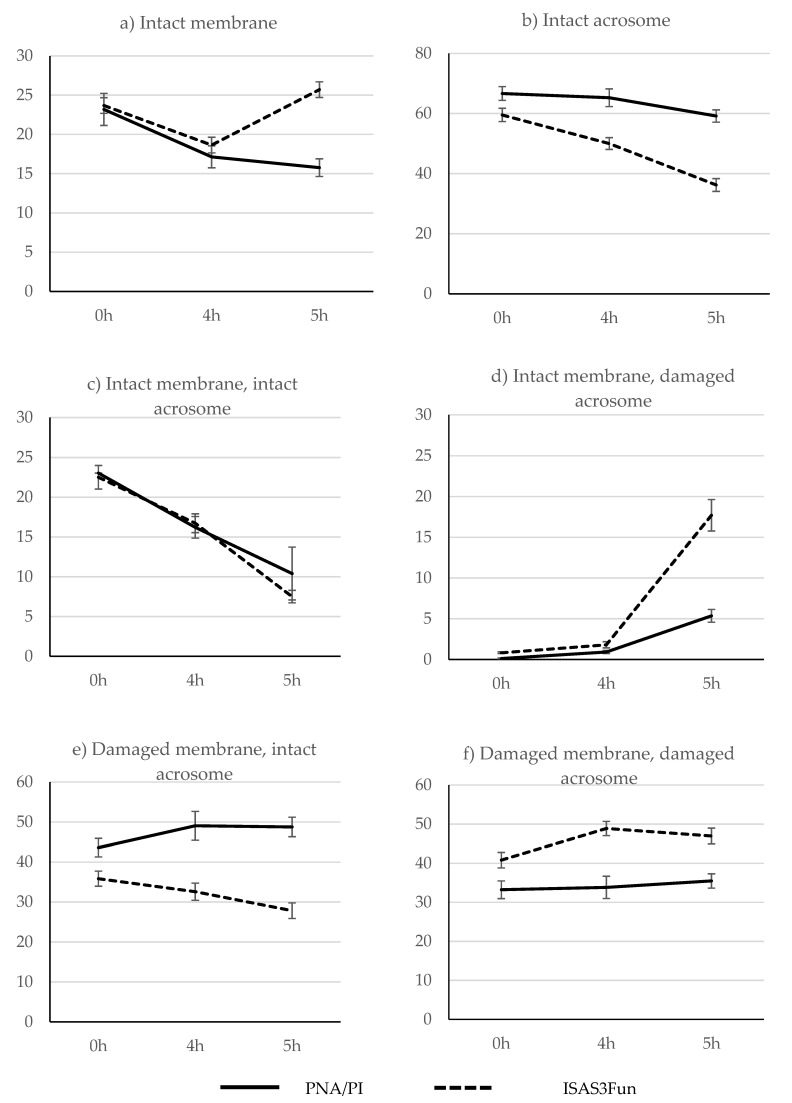
Time evolution of sperm membrane and acrosome integrity using the PNA/PI and the ISAS3Fun fluorescence methods after capacitation (4 h) and ionomycin (5 h) treatment of frozen/thawed bull semen samples (**a**–**f**).

There was a reduction in sperm motility between 0 h (35.88% ± 3.50) and 4 h (27.86% ± 3.25) of incubation, and most spermatozoa were immotile at 5 h. The main model of the GLM repeated measures analysis of factors affecting sperm membrane and acrosome integrity is depicted in Table 4. There was a significant effect of time, time x fluorescent method and the fluorescent method used. These effects are shown in Figure 2. The treatment induced a slight decrease in plasma membrane integrity between 0 h and 4 h of incubation under capacitating conditions, but sperm viability was preserved afterwards, as determined using both fluorescence combinations (Figure 2a). The fluorescent methods clearly differed in the results of acrosome integrity; the percentage of spermatozoa with intact acrosomes was higher using the PNA/PI combination than with the ISAS3Fun method for all incubation periods (Figure 2b). Using phase contrast microscopy, it was observed that spermatozoa without a clearly distinguishable acrosome were not stained with the PNA, generating false negatives that led to an underestimation of the extent of acrosomal damage. Furthermore, using this technique, the reduction in acrosome integrity was only evident at 5 h, while a clear reduction was observed at 4 h with the ISAS3Fun method, followed by a more pronounced acrosomal loss at 5 h after ionomycin treatment (Figure 2b).

In the fluorescence sperm subpopulations, spermatozoa with damaged acrosomes and intact plasma membranes (DAIM) were barely observed at 0 h, then increased slightly at 4 h, and finally increased noticeably at 5 h (Figure 2d). This increase was significantly greater using the ISAS3Fun method than with the PNA/PI combination. In contrast, the proportion of spermatozoa with intact acrosomes and damaged plasma membranes (IADM) was much higher for PNA/PI than for the ISAS3Fun method for all incubating periods and only decreased with the latter method over time (Figure 2e).

## 4. Discussion

Male fertility assessment based on test inseminations is expensive and time consuming [10], and it would be useful to have simple in vitro test for predicting potential bull fertility. In this very restrictive study, in which only one random ejaculate from each bull was used, only the results of the ISAS3Fun technique were significantly related to bull fertility. Spermatozoa from bulls with superior field fertility displayed increased acrosomal integrity and head width. In all likelihood, the repeated analysis of more ejaculates would increase the chance of finding associations between in vitro sperm quality and bull fertility. However, although such relationships were certainly less likely to be observed when using a single ejaculate, the fact that they were found could indicate a consistent relationship with fertility that should be confirmed by further studies. In agreement with our study, Bernecic et al. [15] have described that acrosome integrity, together with viability, were the only sperm attributes that were significantly different between high- and low-fertility bulls. Unlike our work, the evaluation of the acrosome integrity in this study was carried out using PNA, which showed a lower sensitivity than that of the ISAS3Fun technique.

In porcine, it was observed that the number of spermatozoa with reacted acrosomes after capacitation was correlated with prolificacy, and the difference in the rate of reacted acrosome sperm before and after capacitation was correlated with fertility [16]. Therefore, an accurate assessment of the acrosome structure is essential in order to acquire new knowledge of acrosomal exocytosis and to predict the fertilising ability of sperm.

In a previous study in rams, we found clear differences in motility and plasma membrane integrity between high- and low-fertility males, which were not observed in this study of bulls. Unlike in rams, AI in cows is highly efficient, with the semen deposited deeply into the uterus. Consequently, spermatozoa might not have difficulties reaching the site of fertilisation; therefore, high motility and viability might not be as restrictive as in the ewe. Another possible explanation for the differences between these two studies is that the experimental design was different. In the study of rams, fresh semen samples of only eight rams with extreme fertility (four high and four low) were checked weekly during six consecutive weeks. The analysis of frozen/thawed doses of a single ejaculate per bull and the selection of more males per fertility group in the current study would probably reduce the chances of finding differences in more sperm quality parameters. In fact, Bernecic et al. [15] observed that both viability and acrosome integrity could serve as bull fertility biomarkers in the field, while Kjaestad et al. [17] described an association between sperm motility and velocity with bull fertility.

For the establishment of a viable pregnancy, spermatozoa must reach the site of fertilisation, then penetrate and fertilise the oocyte, and, finally, produce a viable embryo. Acrosomal exocytosis is a crucial and irreversible process key in ovum fertilisation that occurs when the acrosomal outer membrane fuses with the plasma membrane at multiple points, releasing proteolytic enzymes [18,19]. Until a few years ago, it was thought that a sperm that had suffered from acrosomal exocytosis could not penetrate the zona pellucida. However, recent studies have orchestrated new scenarios, since it has been observed that spermatozoa that had previously undergone the acrosome reaction were able to penetrate the zona pellucida [18].

Given the difficulty in clearly discerning the sperm acrosome in most animal species, its evaluation is usually limited to research or to occasional studies of sperm quality. In a recent work, we described a simple method that allows the simultaneous visualisation of sperm plasma and acrosomal membrane integrity in bulls by combining the fluorochromes Hoechst 33342, CFDA, and PI (ISAS3Fun) [8]. To facilitate the analysis using this test, a new module was added to the OpenCASA software, which allows for the automated analysis of these sperm quality parameters [12].

CFDA and PI have been combined to assess sperm membrane integrity in several species [11,20,21,22,23,24]. This fluorochrome combination allows the acrosomes of spermatozoa with a damaged plasma membrane to be distinguished, whose red nuclei (PI stained) contrast with the green acrosome (carboxyfluorescein stained) [11,20]. However, live spermatozoa accumulated carboxyfluorescein throughout both the head and the flagellum, and the acrosome is not clearly distinguishable [20,25]. In a previous study [8], we added a third fluorochrome to this combination, Hoechst 33342, allowing the discrimination of acrosomal integrity both in live and dead spermatozoa (ISAS3Fun). The staining pattern of the ISAS3Fun method is opposite to that of the lectins. Only sperm with intact acrosomes contain esterases able to hydrolyse CFDA to carboxyfluorescein, emitting an intense green fluorescence. This fluorochrome has a low molecular weight and is therefore rapidly lost after damage is sustained by the acrosomal membrane, avoiding false negatives. As a consequence, the ISAS3Fun method seems particularly sensitive for the evaluation of acrosomal integrity in bulls. Even in frozen/thawed semen samples (0 h in Experiment 2), the PNA/PI combination underestimated the extent of acrosomal damage when compared to the ISAS3Fun kit.

During sperm capacitation, hyperpolarisation of the plasma membrane occurs, and this hyperpolarisation has been attributed to an increase in calcium during acrosomal exocytosis [18]. Calcium ionophores have long been known to increase intracellular calcium and induce acrosomal exocytosis in mammalian sperm [26]. In the present study, the calcium ionophore challenge validated the use of the ISAS3Fun method to quantify the acrosome integrity in bulls, showing higher sensitivity than traditional PNA/PI staining. In agreement with the findings of other authors [5], it seems that lectin-binding sites disappear after extensive acrosomal damage, producing false negatives that cause an underestimation of the degree of acrosomal injury. Consequently, the fluorescent methods have clearly differed in their results of acrosome integrity, such that the percentage of spermatozoa with intact acrosomes was higher using the PNA/PI combination than with the ISAS3Fun method in most cases. Furthermore, the differences increased with time, particularly after the ionomycin treatment, likely because of the presence of a higher number of spermatozoa with extensive acrosomal loss.

The study of the evolution of the fluorescence sperm subpopulations allowed us to clarify the effect of the treatments. Live spermatozoa showed a clear induced acrosome reaction, with extensive acrosomal damage at 5 h after calcium ionophore was added. This evolution was more clearly appreciated when using the ISAS3Fun combination, with a sudden increase in the proportion of live spermatozoa with damaged acrosomes after ionomycin treatment. The evolution of dead spermatozoa was less evident, showing a reduction in spermatozoa with intact acrosomes when using the ISAS3Fun method, while the proportion of this subpopulation even increased with time when using PNA/PI, likely because the number of spermatozoa with complete acrosomal loss was higher in dead spermatozoa.

Surprisingly, the induction of the acrosome reaction with ionomycin caused an apparent increase in spermatozoa with intact plasma membranes when using the ISAS3Fun kit (Figure 2a). We repeated the experiment several times and observed this increase repeatedly. There is also no clear correspondence between the decrease in the IADM subpopulation (Figure 2e) and the expected increase in the DADM subpopulation (Figure 2f) between 4 h and 5 h. Both events may be related to the increase in the DAIM subpopulation (Figure 2d). At 4 h, we observed the presence of spermatozoa with intact acrosomes and partial permeability to the propidium iodide, which stained red only in the head base, and they were classified as IADM. It is possible that these spermatozoa with intact acrosomes and only partially damaged plasma membranes were still capable of experiencing the acrosomal reaction. At 5 h, we observed a clear increase in the spermatozoa with increased fluorescence intensity at the head and flagellum using the ISAS3Fun kit (IFI subpopulation described in [8], data not shown). Given that the acrosome and the nucleus of most of these spermatozoa were not distinguishable, they were classified as DAIM spermatozoa. However, the increase in fluorescence intensity associated with the acrosome reaction could have hidden the partial staining of the nucleus with propidium iodide, and, therefore, these sperm could have damaged plasma membranes. Despite this, given that the proportion of the IFI sperm subpopulation in the freshly thawed semen is very low, this observation does not seem to limit the options of the ISAS3Fun method to predict potential bull fertility.

## 5. Conclusions

In conclusion, the simultaneous assessment of sperm plasma membranes and acrosome integrity with the ISAS3Fun method seems to have a higher ability to discriminate between high- and low-fertility bulls than a conventional in vitro sperm quality test. Moreover, the use of this technique to quantify acrosome integrity was validated in bulls, with a higher sensitivity than traditional PNA/PI staining.

## Figures and Tables

**Table 1:** Differences in sperm quality parameters between high- and low-fertility bulls. Data are represented as mean ± S.E.M.

Sperm Parameter	High Fertility	Low Fertility
Acrosome integrity (%)	46.20 ± 2.87	35.14 ± 4.43
IADM (%)	24.84 ± 3.14	14.20 ± 2.25
Head width of IAIM (µm)	5.38 ± 0.05	5.25 ± 0.03

IADM: spermatozoa with an intact acrosome and a damaged plasma membrane; IAIM: spermatozoa with an intact acrosome and plasma membrane. The sperm subpopulations were obtained using the ISAS3Fun fluorescence method. The three sperm quality parameters were significantly different between high- and low-fertility groups at *p* < 0.05.

**Table 2:** Discriminant classification matrix showing Fisher’s linear discriminant functions.

	Coefficient of Function of Classification	
	High Fertility	Low Fertility
IADM	−4.907	−5.268
Head width of IAIM (µm)	111.692	96.572
Acrosome perimeter of IAIM (µm)	187.255	195.212
Constant	−1974.853	−2035.336

Values obtained by linear stepwise discriminant analysis. IADM: spermatozoa with an intact acrosome and a damaged plasma membrane; IAIM: spermatozoa with an intact acrosome and plasma membrane. The sperm subpopulations were obtained using the ISAS3Fun fluorescence method.

**Table 3:** Percentage of sperm samples assigned to each fertility group by discriminant analysis.

	Number Allocated to Group by Discriminant Analysis	
	High Fertility	Low Fertility
High fertility	9	1
Low fertility	1	9

90.0% of the samples were classified correctly.

**Table 4:** Main model of the GLM repeated measurement analysis for factors affecting evolution of sperm quality variables over time using frozen/thawed bull semen (*n* = 14).

Subject Effects	Factor	IM	IA	IAIM	DAIM	IADM	DADM
Within	Time	0.0240	<0.0001	<0.0001	<0.0001	0.3340	0.0100
	Time × method	<0.0001	<0.0001	0.3780	<0.0001	<0.0001	0.2050
Between	Method	<0.0001	<0.0001	0.486	<0.0001	<0.0001	<0.0001

IM: spermatozoa with an intact plasma membrane; IA: spermatozoa with an intact acrosome; IAIM: spermatozoa with an intact acrosome and plasma membrane; DAIM: spermatozoa with a damaged acrosome and an intact plasma membrane; IADM: spermatozoa with an intact acrosome and a damaged plasma membrane; DADM: spermatozoa with a damaged acrosome and plasma membrane.

## Data Availability

The data are not publicly available due to them containing information that could compromise research.
